# Enhanced early visual processing in response to snake and trypophobic stimuli

**DOI:** 10.1186/s40359-018-0235-2

**Published:** 2018-05-02

**Authors:** Jan W. Van Strien, Manja K. Van der Peijl

**Affiliations:** Department of Psychology, Education and Child Studies, Erasmus University Rotterdam, PO Box 1738, 3000 DR Rotterdam, The Netherlands

**Keywords:** EEG/ERP, early posterior negativity (EPN), Trypophobia, Snake detection, Phylogenetic fear, Evolution

## Abstract

**Background:**

Trypophobia refers to aversion to clusters of holes. We investigated whether trypophobic stimuli evoke augmented early posterior negativity (EPN).

**Methods:**

Twenty-four participants filled out a trypophobia questionnaire and watched the random rapid serial presentation of 450 trypophobic pictures, 450 pictures of poisonous animals, 450 pictures of snakes, and 450 pictures of small birds (1800 pictures in total, at a rate of 3 pictures/s). The EPN was scored as the mean activity at occipital electrodes (PO3, O1, Oz, PO4, O2) in the 225–300 ms time window after picture onset.

**Results:**

The EPN was significantly larger for snake pictures than for the other categories, and significantly larger for trypophobic pictures and poisonous animal pictures than for bird pictures. Remarkably, the scores on the trypophobia questionnaire were correlated with the EPN amplitudes for trypophobic pictures at the occipital cluster (*r* = −.46, *p* = .025).

**Conclusions:**

The outcome for the EPN indicates that snakes, and to a somewhat lesser extent trypophobic stimuli and poisonous animals, trigger early automatic visual attention. This supports the notion that the aversion that is induced by trypophobic stimuli reflects ancestral threat and has survival value. The possible influence of the spectral composition of snake and trypophobic stimuli on the EPN is discussed.

## Background

People may experience discomfort or aversion when seeing images of clusters of circular objects in proximity to each other, such as honeycombs or seed heads of the lotus flower. This irrational fear of holes or “trypophobia” has been documented only recently in the psychological literature [1, 2], yet has already been the topic of numerous current follow-up studies (e.g, [3–7]). Trypophobia is clearly manifest in 15% of the general population, but nonphobic individuals still rate trypophobic pictures as being less comfortable to view when compared to control pictures [2]. Here, we will use the term “trypophobic” to indicate the potentially aversive visual characteristics of pictures containing clusters of holes. By using this term, we do not suggest that the visual characteristics of these pictures are sufficient for inducing a phobic reaction in most individuals. Individual proneness to trypophobia can be assessed with a symptom scale developed by Le et al. [1], [see Method section for a description]. Trypophobia proneness does not correlate with trait anxiety [1, 6], but appears to be associated with core disgust sensitivity, personal distress, and proneness to visual discomfort [5].

Cole and Wilkins [2] noted the visual nature of trypophobia and performed a spectral analysis on trypophobic and control images. Compared to the control images, the trypophobic images had an excess of contrast energy at midrange spatial frequencies. Further, they analyzed images of the ten most poisonous animals, and images of snakes and spiders. As with the trypophobic images, these images showed relatively high contrast at midrange spatial frequencies. The origin of the trypophobic aversion is therefore thought to be based on its survival value: the visual characteristics of trypophobic stimuli are also found in many highly poisonous animals, and may be triggering automatic threat responses in the brain. Interestingly, a recent study [6] found higher electrodermal responses when participants were viewing trypophobic images compared to control images, which indicates a heightened fear response to trypophobic stimuli. Like fears and phobias towards phylogenetically threatening stimuli such as snakes and spiders, the aversion toward clusters of holes may reflect an evolved preparedness to acquire fear of ancestral threats [8].

Previous studies [9–14] have established that the early posterior negativity (EPN) is highly responsive to phylogenetic fear stimuli. The EPN is an event-related potential (ERP) that reflects early automatic processing of emotionally significant visual information. The EPN is most noticeable at lateral occipital electrodes between 225 and 300 ms after stimulus onset [15]. The EPN indexes ‘natural selective attention’ [16] and the EPN amplitude is amplified by stimuli of evolutionary significance [17]. Given the assumed survival value of trypophobic stimuli, we expected trypophobic stimuli to evoke larger EPN amplitudes than nontrypophobic stimuli. The EPN is often recorded while using a rapid serial visual presentation (RSVP) paradigm. With the RSVP paradigm, a continuous stream of emotional and neutral pictures is presented at a rate of several (typically three) pictures per second to participants who are passively viewing. The RSVP paradigm makes good evolutionary sense because it requires the rapid processing of emotional stimuli under a high processing load [18].

Employing RSVP, we here compare the EPN responses to trypophobic pictures and to poisonous animal pictures with the EPN responses to snake pictures, which in our previous research elicited the highest EPN amplitudes, and to bird pictures, which elicited the lowest EPN amplitudes [12, 14]. Snake pictures and bird pictures thus serve in the present research as reference conditions for the typical EPN responses to phylogenetic threatening and non-threatening stimuli, respectively.

The strongly enhanced EPN amplitudes in response to snake pictures [9, 11–14] have been taken as support for Isbell’s snake detection theory (SDT) [19, 20], which states that the predatory pressure of snakes on primate evolution caused changes in the primate visual system favoring individuals with better ability to visually detect these often hidden and motionless animals. Further support for Isbell’s theory is found by neurophysiological research in macaques that has demonstrated the existence of pulvinar neurons that respond selectively faster and stronger to snake stimuli than to monkey face and hand stimuli [21, 22]. These pulvinar neurons may be part of a feedforward pathway that facilitates processing in the visual cortex [23, 24].

Given the hypothesized survival value of trypophobic pictures and pictures of highly poisonous animals, we would expect in any case larger EPN amplitudes in response to these categories than to bird pictures. We have no clear hypothesis regarding the difference between the EPN amplitudes in response to trypophobic and poisonous animal pictures on the one hand and snake pictures on the other hand. The research of Cole and Wilkins [2] demonstrated that snake pictures, like trypophobic stimuli, had an excess of contrast energy at midrange spatial frequencies. This could implicate comparably enhanced EPN amplitudes to these three categories. The SDT however, proposes that the robust EPN snake effect is specific to the visual perception of snakes and not to the visual perception of other poisonous animals [19, 20]. For that reason, larger EPN amplitudes in response to snake pictures than in response to trypophobic and poisonous animal pictures may be expected.

We further explored whether the individual degree of trypophobia proneness, as measured by a symptom scale, was associated with the EPN amplitude in response to trypophobic pictures.

## Method

### Participants

Twenty-four Dutch university students (12 men, 12 women) with normal or corrected-to-normal vision participated for course credits. Ages ranged from 18 to 26 years, with a mean age of 20.38 years. The study was conducted in accordance with the Declaration of Helsinki and approved by the Ethics Committee of the Department of Psychology, Education and Child Studies of the Erasmus University Rotterdam. All participants provided written informed consent. 

### Questionnaires

Prior to the experimental run, the participants rated their fear of holes by means of the Trypophobia Questionnaire (TQ; Le et al., 2015). The TQ contains 17 items regarding the most common symptoms as a result of viewing trypophobic images, such as “feel uncomfortable or uneasy” and “feel sick or nauseous”. We showed the participants a sheet with the 10 trypophobic pictures (in a 2 by 5 array) that were used in the experiment and asked them to rate the severity of the 17 TQ symptoms when looking at this sheet. These symptoms were rated on a 5-point Likert scale ranging from 1 (not at all) to 5 (extremely), with possible total TQ scores ranging from 17 to 85.

In addition, participants rated their fear of snakes on a 15-item questionnaire (see, Van Strien, Eijlers, et al., 2014) with a 4-point Likert scale ranging from 0 (not true) to 3 (very true), with possible total scores ranging from 0 (no fear) to 45 (very high fear). For this questionnaire, no pictures were shown.

Following the experimental run, participants performed a computerized Self-Assessment Manikin (SAM) questionnaire [25] regarding valence and arousal ratings of all pictures on a 9-point scale. For each consecutive picture, the participants first rated valence and then arousal. The order of pictures was random for each participant.

### Stimuli and procedure

**Fig. 1:**
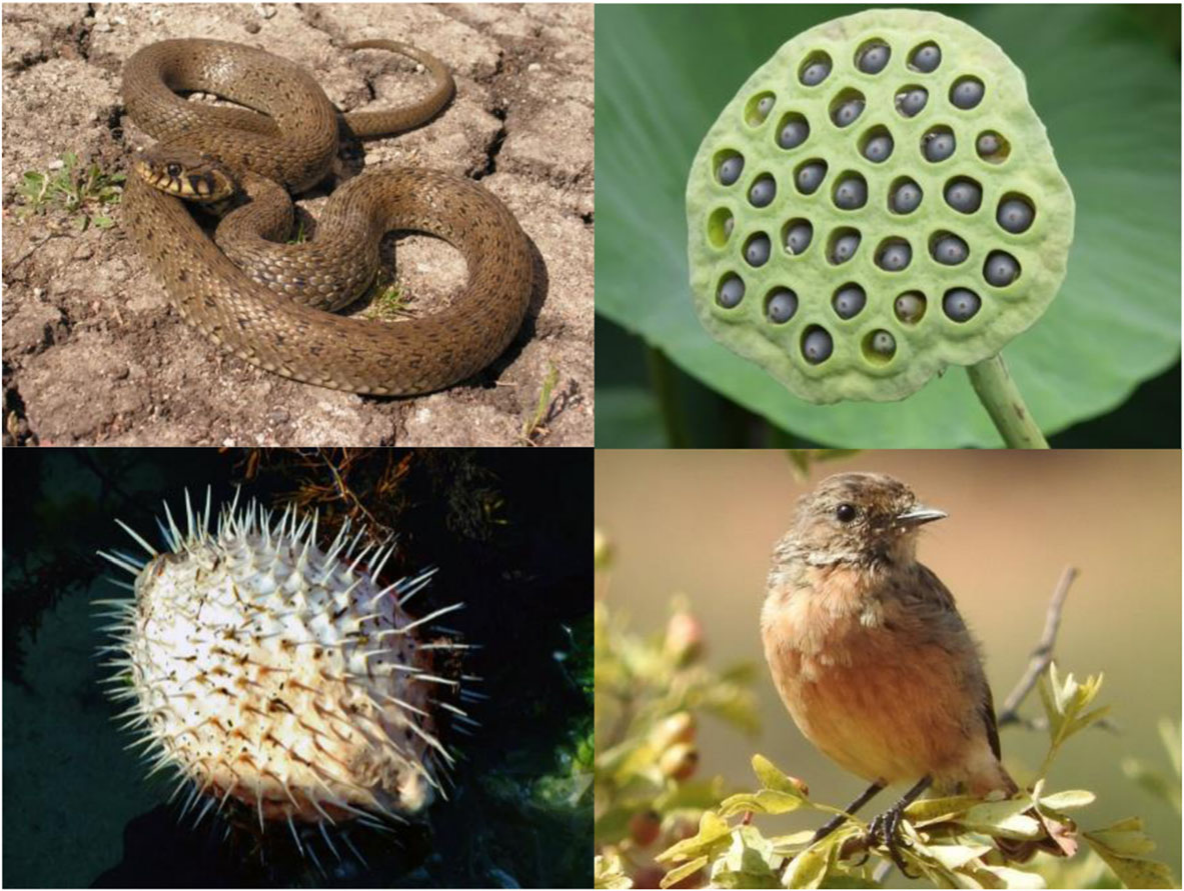
Illustrative examples of snake, trypophobic, poisonous animal, and small bird stimuli. The depicted photographs are public domain (pixabay.com); they are similar to the stimuli used in the present research

Participants were seated in a dimly-lit room and were told to attentively watch the random and continuous RSVP of 450 snake pictures, 450 pictures of trypophobic objects, 450 pictures of poisonous animals, and 450 pictures of small birds. These four different stimulus categories were not explicitly mentioned to the participants. The random presentation ensured that each stimulus category in the RSVP stream was preceded by all other categories in an equal fashion, balancing any carry-over effects. The presentation rate was 3 pictures per second, with no blank between pictures. For each stimulus category, there were 10 different pictures that were shown 45 times Snake and bird pictures were obtained from previous studies [12, 13]. Pictures of poisonous animals were obtained from various internet sites. The 10 poisonous animals were the blue-ringed octopus, the box jellyfish, the Brazilian wandering spider, the death stalker scorpion, the marbled cone snail, the golden poison frog, the puffer fish, the stone fish, the Portuguese man-of-war and the Sydney funnel-web spider. The first eight animals in this list were also in the list of the 10 most poisonous animals employed by Cole and Wilkins (2013). Because in our research snakes were a separate stimulus category, we replaced two snakes from Cole and Wilkins’ list with two other poisonous animals. Each animal picture showed a complete specimen against a natural background (see Fig. 1). Trypophobic images were taken from various websites that were found with Google Search using “trypophobia” as a search term. The trypophobic picture set included barnacles, lotus seeds, pepper seeds and membrane, sliced cantaloupe, coral, honeycomb, and several spongy structures such as in sandstone. All picture sets used in the current research are available from the corresponding author.

The pictures were shown at a distance of 120 cm on a PC monitor with a diagonal of 51 cm and a resolution of 1024 × 768 pixels. Pictures were displayed against a medium grey background and had a size of 600 × 450 pixels, which resulted in a visual angle of 11.40° × 8.55°.

### EEG recording and analysis

**Fig. 2:**
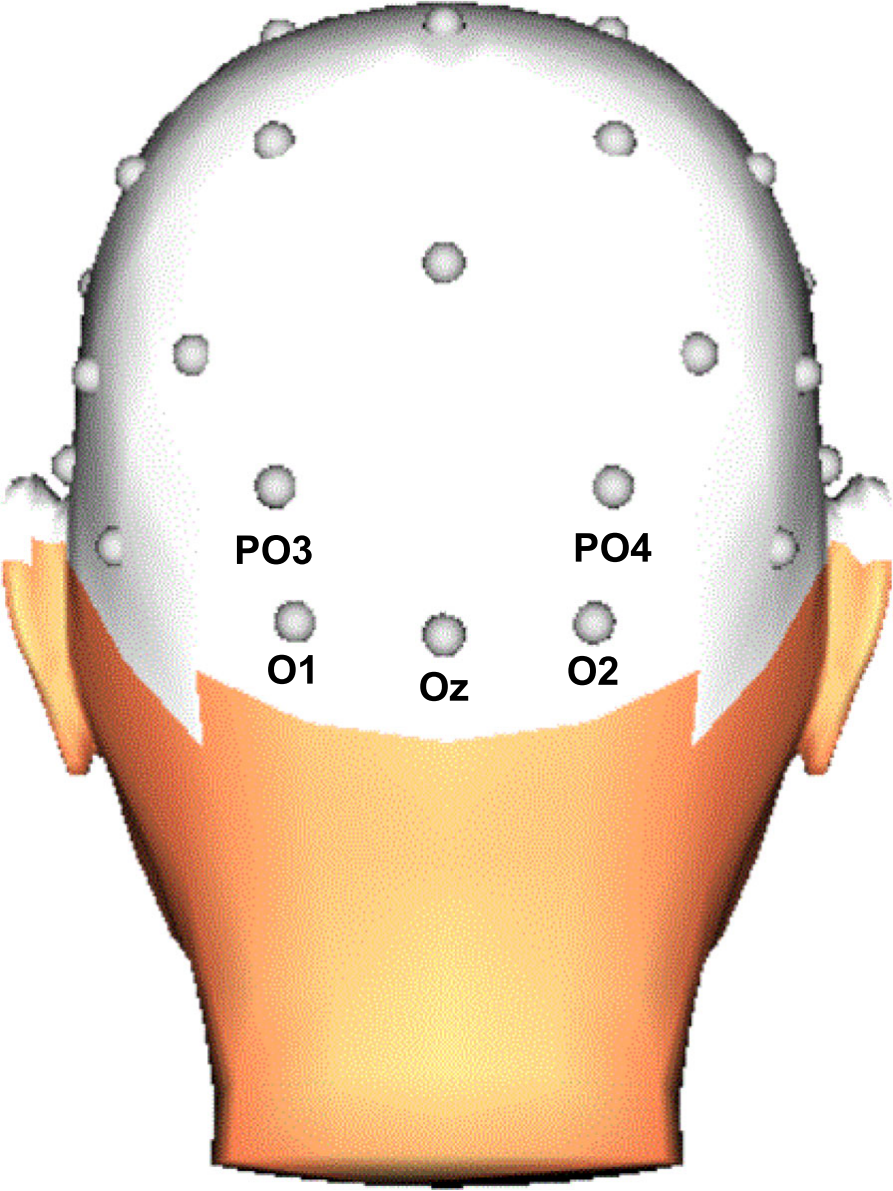
Diagram of the EEG electrodes included in the statistical analysis

EEG recording was done with a BioSemi Active-Two amplifier from 32 scalp sites with active Ag/ AgCl electrodes mounted in an electrode cap (10–20 system). Electrooculogram (EOG) activity was recorded with active electrodes placed above and beneath the left eye, and with electrodes placed at the outer canthus of each eye. The EEG and EOG signals were digitized with a sampling rate of 512-Hz and 24-bit A/D conversion. Offline, the EEG signals were referenced to an average reference. All signals were filtered with a band pass of 0.10–30 Hz (phase-shift-free filter, 24 dB/Oct). Horizontal and vertical eye movements were corrected using the Gratton and Coles algorithm [26]. ERP epochs were extracted with a 380-ms duration and beginning 50 ms before stimulus onset. The ERP signals were computed relative to the mean of this 50-ms prestimulus baseline period. For each participant and each condition, average ERPs were defined. Epochs with a baseline-to- peak amplitude difference larger than 100 μV on any channel were omitted from averaging. In each condition, the mean percentage of valid epochs at analysis-relevant electrodes was more than 99% (with 450 presentations per condition). Similar to previous research, the EPN was scored at occipital electrodes (O1, O2, Oz, PO3, and PO4; see Fig. 2) and was measured as the mean amplitude of the 225–300 ms time window after stimulus onset (e.g., Van Strien et al., 2016; Van Strien, Eijlers, et al., 2014; Van Strien, Franken, et al., 2014).

### Spatial frequency analysis

As a post-hoc check, the spectral compositions of the pictures that were used in the present tasks, were measured by employing a discrete wavelet analysis on each picture, using the Matlab routines *freqspat.m* and *freqspat_gui.m* as described and provided by Delplanque et al. [27]. With discrete wavelet analysis, the picture is decomposed in eight independent spatial frequency bands of which the energy is determined. If a picture contains much small features (i.e., details), the analysis will result in higher energy for high spatial frequencies. If a pictures contains much large features, the analysis will result in higher energy for low spatial frequencies. We measured spatial frequencies in cycles per degree of visual angle (cpd), which represents the frequencies perceived by an observer and depends on the distance between stimulus and observer. It should be noted that the spatial frequency analysis was done after picture selection and did not play a role in this selection.

### Statistical analyses

For the valence and arousal ratings, repeated-measures analyses of variance (ANOVAs) were employed with stimulus category (snakes, trypophobic objects, poisonous animals, birds) as factor. For the EPN components, a repeated-measures ANOVA was conducted, with stimulus category (snakes, trypophobic objects, poisonous animals, birds) and electrode (O1, Oz, O2, PO3, PO4) as factors. When appropriate, Greenhouse-Geisser correction was applied. To explore the relationship between reported trypophobia proneness and EPN amplitudes in response to trypophobic pictures, and between snake fear and EPN amplitudes in response to snake pictures, we calculated the Pearson correlations between questionnaire scores and EPN amplitudes for trypophobic and snake stimuli, respectively. To reduce the number of correlations, we employed one occipital cluster (comprising O1, O2, Oz, PO3, and PO4) for the EPN amplitude measures. Possible differences in spatial frequency power between the four stimulus categories were tested using separate Kruskal-Wallis nonparametric tests for each spatial frequency band.

## Results

### EPN

**Fig. 3:**
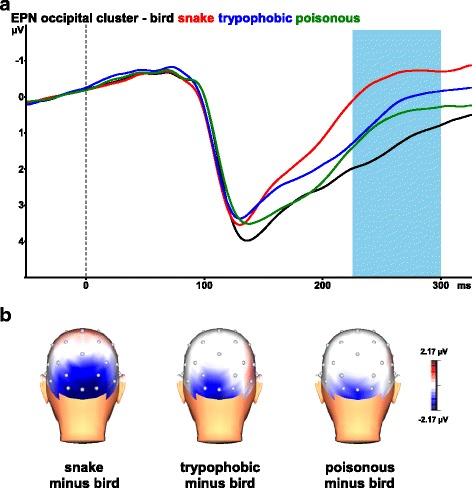
**a** The early posterior negativity (EPN) in response to snake (red line), trypophobic (blue line), poisonous animal (green line) and bird pictures (black line) across the five occipital electrodes (O1/2, Oz, PO3/4). The depicted waveform for each condition is the grand average of 24 participants with approximately 450 epochs per participant. Negativity is up. **b** Topographic maps of the differences in EPN mean amplitudes (225–300 ms) between snake vs. bird pictures (left), trypophobic vs. bird pictures (middle), and poisonous animal vs. bird pictures (right)

The ANOVA revealed a significant main effect of stimulus category, *F*(3,69) = 25.28, *ɛ* = 0.765, *p* < 0.001, *η*^*2*^_*p*_ = .524. Bonferroni-corrected pairwise comparisons revealed that the EPN was significantly more negative for snake pictures than for the other categories (all *p*-values < .001, see Fig. 3a for the mean ERPs across the five occipital electrodes). Trypophobic pictures (*p* = .001) and poisonous animal pictures (*p* = .034) evoked a more negative EPN than bird pictures. No significant difference in EPN amplitude was found between trypophobic pictures and poisonous animal pictures (*p* > .999).

The ANOVA further revealed a significant interaction of stimulus category and electrode, *F*(12, 276) = 9.15, *ε* = .445, *p* < .001, *η*^*2*^_*p*_ = .285. As can be seen in Fig. 3b, the enhanced EPN was more widespread (including PO3 and PO4) for snake pictures than for trypophobic and poisonous animal pictures. To further evaluate the significant interaction of stimulus category and electrode, the stimulus category effects were tested at single electrodes. These analyses revealed significant stimulus category effects at all included electrodes (all *p*-values < .001). Pairwise comparisons with Bonferroni adjustment for multiple comparisons indicated that, compared to bird pictures, snake pictures evoked larger EPN amplitudes at all included electrodes (all *p*-values < .001). Compared to bird pictures, trypophobic pictures evoked larger EPN amplitudes at PO3, O1, Oz, and O2 electrodes (all *p*-values ≤ .038). Compared to bird pictures, poisonous animal pictures evoked larger EPN amplitudes at O1 and Oz electrodes (both *p*-values ≤ .011).

### TQ and snake fear scores

The mean TQ score was 21.04 (SD = 5.18; range 17–36), indicating a relatively low trypophobic repulsion level in the present sample. Remarkably, the TQ scores were correlated with the EPN occipital cluster amplitudes in response to trypophobic pictures (*r* = −.46, *p* = .025), with participants that experienced higher aversion to these stimuli showing larger EPN amplitudes.

The mean snake fear score was 11.75 (*SD* = 8.25; range 2–34); there was no significant correlation between the fear ratings for snakes and the EPN occipital cluster amplitude measure in response to snake pictures (*r* = .02).

### Valence and arousal ratings

The mean SAM valence and arousal ratings for snake pictures, trypophobic pictures, poisonous animal pictures, and small bird pictures are given in Table 1. The main stimulus category effects were significant for both valence, *F*(3,69) = 18.19, *ε* = .649, *p* < .001, *η*^*2*^_*p*_ = .442, and arousal, *F*(3,69) = 14.80, *ε* = .591, *p* < .001, *η*^*2*^_*p*_ = .391. Bonferroni-corrected comparisons revealed that that bird pictures were rated as more pleasant than pictures of trypophobic objects, snakes, and poisonous animals (all *p*-values <.001).

**Table 1:** Participants’ mean arousal and valence ratings (and standard deviations)

Stimulus category	Valence (SD)	Arousal (SD)
Snakes	4.68 (1.92)	3.47 (1.97)
Trypophobic objects	4.30 (1.25)	2.23 (1.55)
Poisonous animals	4.53 (1.35)	3.63 (1.50)
Small birds	6.53 (1.21)	1.58 (.94)

Note. Valence and arousal ratings are based on a rating scale from 1 to 9

Pictures of poisonous animals were rated as more arousing than both bird pictures and trypophobic pictures (both *p*-values < .009). In addition, snake pictures were rated as more arousing than bird pictures (*p* < .001). There were no difference in valence and arousal ratings between remaining stimulus category pairs (all *p*-values > .398).

### Spatial frequency analysis.

**Fig. 4:**
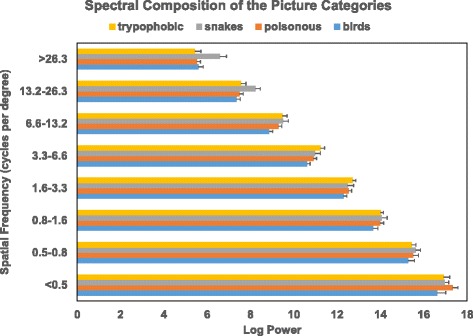
Mean energy for each frequency band as a function of picture category. Error bars depict standard error of means. Frequency bands are expressed in cycles per degree of visual angle. High spatial frequencies are on top

Kruskal-Wallis tests revealed significant category effects for the two highest spatial frequency bands (> 26.3 cpd, *p* = .007; 13.2–26.3 cpd, *p* = .029). From Fig. 4 it can be seen that snake pictures clearly exhibit higher energy for these frequency bands when compared to the other categories. Although the energies for the midrange spatial frequency bands (1.6–3.3 cpd and 3.3–6.6 cpd) were slightly higher for trypophobic pictures compared to the other categories, there were no further significant category effects (all *p*-values > .067).

## Discussion

Using the RSVP paradigm, we compared the EPN responses to trypophobic and to poisonous animal pictures with the EPN responses to snake pictures, which in previous research elicited the highest EPN amplitudes, and to bird pictures, which elicited the lowest EPN amplitudes. Given the potential phylogenetic threat of trypophobic objects and poisonous animals, we expected larger EPN amplitudes in response to trypophobic pictures and pictures of poisonous animals than to pictures of birds. The EPN results were in line with our expectations, with the EPN being equally enhanced for trypophobic objects and poisonous animals when compared to birds. Yet, as in previous research [11–13], snake pictures elicited the largest EPN when compared to the three other stimulus categories.

The equally enhanced EPN amplitudes in response to trypophobic and poisonous animal pictures indicate that both stimulus categories attract early visual attention to the same extent. As the EPN is thought to reflect natural selective attention to stimuli of evolutionary significance [16, 17], this outcome may support the notion that the origin of trypophobic aversion is based on its survival value, with visual characteristics akin to that of poisonous animals triggering automatic threat detection responses in the brain [2].

However, when compared to trypophobic and poisonous animal pictures, snake pictures elicited even larger EPN amplitudes. This robust EPN snake effect is identical to the results obtained in our previous research involving snake pictures, which all demonstrate the largest EPN amplitudes in response to snake stimuli [11–14]. The large and consistent EPN enhancement in response to snake pictures reflects high early capture of human visual attention by snakes and clearly supports Isbell’s SDT [19, 20]. According to the SDT, snakes have acted during evolution as a selective pressure in the modification and expansion of the primate visual system, resulting in greater visual sensitivity to snakes than to other stimuli. The higher EPN to snake pictures than to trypophobic and poisonous animal pictures could reflect a higher level of phylogenetic threat in case of snakes. As snakes are venomous predators that actively chase and inoculate venom by biting their prey, they were more life-threatening to our ancestors than other poisonous animals, which are only dangerous when touched or ingested.

It should be noted that, in addition to the trypophobic and poisonous animal pictures from the present research, moderate EPN enhancements have been demonstrated in response to a variety of other emotional stimuli, not necessarily representing phylogenetic threat [18]. Therefore, it remains uncertain whether the EPN in response to trypophobic and poisonous animal stimuli is only determined by level of phylogenetic threat.

In the present and previous studies, we employed naturalistic stimuli (i.e., realistic pictures of snakes, trypophobic objects, poisonous animals, and birds). By doing so, we did not control for low-level visual features, such as color, contrast, luminance, and spatial frequency of the pictures, which might influence the EPN. In our research, there is always tension between ecological valid, naturalistic stimuli and “vision-science” stimuli equated for low-level visual characteristics. We here preferred to use ecologically valid stimuli because the low-level features as such may be inherent properties of the fear stimuli and may be important for threat detection. It is obvious that, once the attention-grabbing and ERP boosting effects of naturalistic stimuli are established, it is worthwhile to detect the fundamental visual mechanisms of fear detection and to further study the formal visual characteristics of these threat stimuli. Previous research has indicated that the effects of some low-level features, such as color and luminance, on the EPN in response to snake pictures most probably are marginal. Research employing brightness-equated grayscale pictures [28] or luminance- and contrast-equated color pictures [9] yields EPN snake effects that are highly comparable to the effects that we have found with naturalistic stimuli.

Here we explored the spatial frequency characteristics of the four stimulus categories, because previous research [2] has established an excess of contrast energy at midrange frequencies for trypophobic and poisonous animal stimuli. The range of spatial frequencies for which an excess energy may induce discomfort has been determined to be 1–8 cpd [29]. Although we found slightly higher energies in midrange spatial frequency bands (1.6–3.3 cpd and 3.3–6.6 cpd) for trypophobic pictures compared to the other categories, we found no statistically significant category effects for the energy in midrange frequencies. It should be noted that the failure to find such differences could be due to the small number of pictures in each category, which reduced the power to detect any differences in midrange frequencies.

Our spatial frequency analysis did reveal an excess energy at higher spatial frequencies (> 13.2 cpd) for snake pictures. This finding is in accordance with the results of the spatial frequency analysis by Delplanque et al. [27], which revealed that pictures of snakes from the International Affective Picture System [30] contain significantly more high frequency energy when compared to pictures of other unpleasant animals. This excess of high spatial frequencies may be caused by the typical snake skin scales and scale patterns. Van Strien and Isbell [14] found higher EPN amplitudes in response to close-ups of snake skins than to close-ups of lizard skins and bird feathers. In addition, blurring snake pictures, and thus reducing the higher spatial frequencies, attenuated the EPN amplitudes when compared to non-blurred snake pictures [31]. Future work should determine the specific relationship between EPN amplitude and the energy of high and midrange spatial frequencies.

In our sample, the trypophobia proneness of the participants as assessed by the TQ was rather low. Le et al. [1] have suggested a TQ score above 31 as a criterion to determine the existence of a real phobia. In our sample only two one out of 24 participants (8%) met this criterion, which is lower than the estimated 15% of the general population [2]. Although in the present sample the reported discomfort in response to trypophobic objects was modest, and arousal scores were low, the EPN was clearly enhanced in response to the trypophobic pictures. Moreover, the EPN amplitudes elicited by these pictures correlated significantly with the TQ scores, with participants with higher TQ scores showing larger EPN amplitudes. This association suggests that individuals are adequately aware of their physical responses to trypophobic stimuli and that the degree of these responses is reflected in the EPN.

The EPN amplitudes elicited by snake pictures did not correlate with snake fear scores. This is in agreement with our previous studies, in which significant correlations for snake fear and EPN amplitude in response to snake pictures were not found either [11–13]. This lack of an association may be due to the fact that most of our participants probably never have engaged snakes in the wild and hence cannot adequately report their actual fear of snakes. This is possibly also reflected in the participants’ relatively low arousal ratings for the snake pictures. The lack of an association between reported snake fear and EPN amplitude is not inconsistent with the SDT, which presumes that the early visual processing of snakes is innate and automatic. In several previous studies, we have found an association between reported spider fear and the EPN in response to spider stimuli [12, 32]. Like for the TQ scores in the present research, individuals may be adequately aware of their emotional responses to spiders, which is reflected in the EPN. Whether the supposed individual’s better conscious awareness of emotional responses to trypophobic objects, poisonous animals, or spiders than to snakes is indicative a more non-evolutionary or cultural nature of visual processing awaits further research.

## Conclusion

We employed random RSVP of snake pictures, trypophobic pictures, poisonous animal pictures, and bird pictures, and found that the EPN was larger for snake pictures than for pictures of the other categories. In addition, trypophobic pictures and pictures of poisonous animals resulted in larger EPN amplitudes than bird pictures. The scores on the trypophobia questionnaire were correlated with the EPN amplitudes for trypophobic pictures, suggesting the participants’ adequate awareness of their physical responses to trypophobic stimuli. The outcome for the EPN indicates that snakes in particular, and trypophobic stimuli and poisonous animals to a lesser extent, trigger early automatic visual attention. This lends support to the notion that the aversion that is induced by trypophobic stimuli reflects ancestral threat and has survival value [2], although the detection of snake stimuli most probably has much larger survival value [19, 20]. The triggering of early automatic visual attention as reflected in the EPN may be based on the spectral composition of the phylogenetic threatening stimuli, with snake stimuli in particular exhibiting an excess energy at high spatial frequencies.

## Abbreviations

EPN: Early posterior negativity
ERP: Event-related potential
RSVP: Rapid serial visual presentation
SAM: Self-assessment manikin
SDT: Snake detection theory
TQ: Trypophobia questionnaire
